# Climate-sensitive health counselling in Germany: a cross-sectional study about previous participation and preferences in the general public

**DOI:** 10.1186/s12889-024-18998-6

**Published:** 2024-06-06

**Authors:** Nicola Krippl, Nikolaus C.S. Mezger, Ina Danquah, Jessica Nieder, Silvan Griesel, Jan Schildmann, Rafael Mikolajczyk, Eva J. Kantelhardt, Alina Herrmann

**Affiliations:** 1 Heidelberg Institute of Global Health, Medical Faculty and University Hospital, Heidelberg University, Im Neuenheimer Feld 130.3, 69120 Heidelberg, Germany; 2 Global and Planetary Health Working Group, Interdisciplinary Center for Health Sciences, Medical Faculty, Martin Luther University Halle-Wittenberg, Magdeburger Straße 8, 06097 Halle (Saale), Germany; 3 Hertz-Chair Innovation for Planetary Health, Center for Development Research (ZEF), Rhenish Friedrich Wilhelm University of Bonn, Genscherallee 3, 53113 Bonn, Germany; 4 Institute for History and Ethics in Medicine, Interdisciplinary Center for Health Sciences, Medical Faculty, Martin Luther University Halle-Wittenberg, Magdeburger Straße 20, 06112 Halle (Saale), Germany; 5 Institute for Medical Epidemiology, Biometrics and Informatics, Interdisciplinary Center for Health Sciences, Medical Faculty of the Martin, Luther University Halle-Wittenberg, Magdeburger Straße 8, 06097 Halle (Saale), Germany; 6 Institute of General Medicine, University Hospital Cologne, Medical Faculty Cologne University, Kerpener Straße 62, 50937 Cologne, Germany

**Keywords:** Patient counselling, Health promotion, Climate change, Physician-patient interaction, Patient preferences, Patient participation

## Abstract

**Background:**

In response to climate change (CC), medicine needs to consider new aspects in health counselling of patients. Such climate-sensitive health counselling (CSHC) may include counselling patients on preventing and coping with climate-sensitive diseases or on leading healthy and climate-friendly lifestyles. This study aimed to identify previous participation in and preferences for CSHC as well as associated sociodemographic and attitudinal factors among the general public in Germany.

**Methods:**

We conducted a cross-sectional study in a population-based online panel in five German federal states (04–06/2022). We performed descriptive statistics and multivariable regression analysis to assess prior participation in CSHC and content preferences regarding CSHC, as well as associations between sociodemographic variables and general preference for CSHC.

**Results:**

Among 1491 participants (response rate 47.1%), 8.7% explicitly reported having participated in CSHC, while 39.9% had discussed at least one CSHC-related topic with physicians. In the studied sample, 46.7% of participants would like CSHC to be part of the consultation with their physician, while 33.9% rejected this idea. Participants aged 21 to 40 years (versus 51 to 60), individuals alarmed about CC (versus concerned/cautious/disengaged/doubtful/dismissive), and those politically oriented to the left (vs. centre or right) showed greater preference for CSHC in the multivariable regression model. Most participants wanted to talk about links to their personal health (65.1%) as opposed to links to the health of all people (33.2%).

**Conclusions:**

Almost half of the participants in this sample would like to receive CSHC, especially those who are younger, more alarmed about CC and more politically oriented to the left. More research and training on patient-centred implementation of CSHC is needed.

**Supplementary Information:**

The online version contains supplementary material available at 10.1186/s12889-024-18998-6.

## Background

Humanity is crossing planetary boundaries by destabilizing natural and social systems upon which human health depends [1–3]. Climate change (CC) is one of these planetary boundaries which poses immediate threats to human health [4–6]. Globally, CC increases the frequency and intensity of extreme weather events, leading to more injuries, deaths, mental health problems and heat-related diseases [4]. Furthermore, climate change impacts the patterns of infectious diseases, increases the risk of food insecurity and has implications for conflict and migration [4]. In Germany, some of the most relevant health risks include heat stress, an extended pollen season, changing patterns of infectious diseases and mental health issues [7–12]. For instance, heat events in the summer of 2018 lead to approximately 8700 heat-related deaths in Germany [13].

In order to cope with climate-sensitive diseases, health systems should become climate-resilient and reduce their own greenhouse gas emissions according to the World Health Organization (WHO). Delivering climate-informed health programs is one of the key elements in the WHO’s operational framework for climate-resilient and low-carbon health systems [14]. As part of this, health professionals can educate patients about health risks of climate change and offer appropriate protective measures [15, 16]. Furthermore, health professionals can integrate advice about healthy and climate-friendly lifestyles into their counselling [17, 18]. For instance, largely plant-based diets and active modes of mobility can promote health and mitigate climate change [19–24].

One recently published concept to cope with climate-sensitive diseases and promote healthy and climate-friendly lifestyles in health systems is climate-sensitive health counselling (CSHC). The aims of CSHC are defined as protecting and promoting public and planetary health, raising knowledge and awareness of CC and health issues, and encouraging climate action and lifestyle change [25]. However, only a few studies have assessed whether CSHC is already being practiced. Boland et al. reported that 10% of US primary care patients had already discussed environmental issues and their effects on health with a physician [26]. In a US paediatric practice (Pennsylvania), 4% of the parents (*n* = 371) reported that their paediatrician had previously discussed global warming during well-child visits [27]. In Germany, a study investigated climate-specific health literacy in outpatient practices: 13.4% of the sample of general practitioners’ patients (*n* = 329) and 15.5% of gynecologists’ patients (*n* = 91) reported receiving climate-specific medical advice [28]. In addition, the lack of knowledge about patients’ preferences for CSHC is a major barrier to its implementation, as identified in studies with physicians [26, 29–31]. In the US paediatric practice mentioned above, 80% of the parents (*n* = 371) (strongly) agreed that providers should discuss the effects of global warming on children’s health during visits [27]. However, there is little other evidence on the preference for CSHC in larger samples. Therefore, it remains unclear whether patients would like to receive CSHC and which preferences they have regarding specific content. This knowledge is important for physicians and potentially other health professionals aiming to tailor CSHC to patients’ preferences.

To assess participation in and preferences for CSHC in a population-based sample in Germany, this study investigated the following research questions: A. What share of participants have already participated in CSHC and related counselling topics? B. What are the general and content-specific preferences for CSHC? and C. What are the possible associations between socioeconomic and attitudinal characteristics and preferences for CSHC?

## Methods

### Study design and sampling

We conducted a cross-sectional study in the population-based online panel HeReCa (short for “Health-Related Beliefs and Health Care Experiences in Germany”) from April to June 2022. In this panel, established in 2020, participants are asked to participate in surveys on health-related aspects four times a year.

A random sample of individuals aged 20 to 79 years was drawn from registration office data from 12 to 15 municipalities from each of the five federal states. The selection of municipalities and the proportion of invited participants should reflect the regional distribution of population density in terms of less densely populated regions, towns and cities. The recruitment and composition of the panel are described in more detail by Klee et al. 2024 [32]. Currently 3163 individuals from five federal states in Germany (Baden-Württemberg, Berlin, North Rhine-Westphalia, Saxony-Anhalt, Schleswig Holstein) are part of the HeReCa panel and were invited to participate in this survey via e-mail. Supplementary Fig. 1.1. provides an overview of the recruitment procedures for the original panel and the present study. With regard to sample size, we aimed to carry out a full assessment of the HeReCa Panel to obtain a good descriptive estimate of previous participation and preferences for CSHC in this sample. For the exploratory linear regression models with a limited number of independent variables we considered the expected sample size of over 1000 to be sufficiently large [33].

### Survey tool

As a literature search did not reveal a validated tool to assess preferences for CSHC, we developed a new questionnaire based on a framework for CSHC counselling [25], further literature [28, 34–36] and other qualitative research on CSHC in patients and physicians in Germany within our working group [18, 37]. The questionnaire was pretested with a paper version (5 respondents) and pilot-tested with an online version (19 respondents); written feedback led to minor changes in the wording of the items. The final questionnaire included 47 items, 12 of which were derived from previously published work. The first part of the questionnaire (15 items) consisted of case-vignettes about climate-sensitive lifestyle counselling rated for acceptability. These 15 items on case vignettes are not part of the analysis in this paper. Table 1 provides an overview of the remaining 32 items relevant for this paper, which are divided into eight sections (A-H) and presented in the original order. The 32-item questionnaire can be found in the supplementary material.

**Table 1:** Structural overview of the questionnaire with the number of items

	Questionnaire section	No. of items
A	Attitudes on climate change (SASSY*)	4
B	Readiness for climate-friendly behaviour	6
C	Prior participation	2
D	Content preferences for CSHC	16
	o D1: Information about CC impacts (in general, heat waves, allergies, infectious diseases)	(4/16)
	o D2: Talking about climate anxiety and climate action	(2/16)
	o D3: Advice on CC adaptation/health protection (in general, heat waves, allergies, infectious diseases)	(4/16)
	o D4: Links of CC with individual and population health	(2/16)
	o D5: Healthy and climate-friendly lifestyles (in general, nutrition, mobility, mental health)	(4/16)
E	Preferred information channels	1
F	General CSHC preference	1
G	Political self-positioning	1
H	Number of Physician visits per year	1
	**Overall**	**32**

*Six Americas Short Survey, SASSY

We assessed the participants’ attitudes towards CC using the Six Americas Short Survey (SASSY) tool [38]. The SASSY is a short version (4 items) of a 36-item instrument validated to assign the respondents to one of six categories regarding CC attitudes (*alarmed*, *concerned*, *cautious*, *disengaged*, *doubtful* and *dismissive*). We translated the original tool into German and applied the associated online tool to segment the sample into six groups [34]. To evaluate participants’ readiness to implement climate-friendly behaviour in their everyday life, we derived six items from a questionnaire on climate-specific health literacy [28]. Regarding their prior participation in CSHC, we asked participants if CC and health topics had ever been addressed in consultations with a physician and adjoined a list on which participants could rate whether predefined issues had been addressed in these consultations.

We assessed the preferences for different CSHC contents on a 5-point Likert scale (*No, not at all/Rather No/Doesn’t matter/Rather Yes/Yes, very much*): for health impacts of CC, we used the phrase “*Would you like your physician to inform you about the health impacts of CC?”* (general item, plus subitems on heat waves, allergies and infectious diseases); for adaptation measures, “[…] *to advise you on how to protect your health from the effects of CC?”* (general item, plus subitems on heat waves, allergies and infectious diseases) and for climate-friendly lifestyle changes “*Would you like to talk to your physician about how you can lead a healthy and climate-friendly lifestyle?*” (general item, plus subitems on nutrition, mobility and mental health). Furthermore, we assessed preferences for talking about fear or concerns regarding the impacts of CC (1 item) and about the possibility of engaging in climate action (1 item). We also assessed preferences for discussing the links between CC, personal health, and population health and preferred information channels for CSHC (multiple answers possible). Then, we asked participants for their general CSHC preference: *“You have now learned about possible topics that can be part of climate-sensitive health counselling. Please answer the following summary question: Would you like topics of climate change and health to be part of the consultation with your physician?”*. We furthermore assessed political self-positioning (scale of 10, from 1 (left) to 10 (right) [35]) and the frequency of physician visits over the last 12 months [36].

### Data analysis

Only participants who completed the last topic-related item (Section F in Table 1) were included in the descriptive analysis, consequently 25 incomplete questionnaires were excluded. We used SPSS V29.0. for the analysis.

To follow research questions A and B, we used descriptive statistics (means, SDs, percentages) to describe the study population’s sociodemographic characteristics and depict their answers to our survey questions. To investigate the internal reliability of the three major topics of CC, namely impacts (D1 in Table 1), adaptation measures (D3), and lifestyle changes (D5), we constructed a composite score from the respective items a-d. Internal reliability was high with Cronbach’s α ranging from 0.88 to 0.91.

For research question C, we ran univariate regressions for all dependent and independent variables to explore the data. We then built a theory-driven multiple linear regression model with three steps in which we regressed general CSHC preference (Section F) onto demographics in step 1, adding socioeconomic characteristics in step 2, and attitudinal variables in step 3 (see Table 5).

13.1% of the cases included in the descriptive analysis had missing data for the regression model. We compared the sociodemographic characteristics of participants with missing data with those without missing data and did not find relevant differences (see Supplementary Table 2.4). Therefore, we applied listwise deletion to the full model, leading to the exclusion of 195 cases (see Suppl. Figure 1.1).

Age, sex, income, education, and SASSY type were treated as categorical variables, whereas political self-positioning was treated as a continuous variable. We used the Variance Inflation Factor (VIF) to check for multicollinearity between the independent variables. As a criterion for statistical significance, we applied a probability level of *p* = 0.05.

## Results

### Sociodemographic characteristics

In total, 1491 participants took part in the survey (response rate of 47.1%). The mean age of the respondents was 55.6 years, 62.9% of participants reported high-level school education, 57% of them were female and 96.1% had seen a physician at least once within the last year. Overall, political self-positioning displayed a slight left tendency (mean 4.4 ± 1.6, scale from 1(left) to 10 (right)). All five federal states were similarly represented. For more information, see Table 2.

**Table 2:** Summary of sociodemographic and attitudinal characteristics

	*n* (total)	*n*	%
**Age (years) mean ± SD**		55.6 ±14.2	
**Age min/max**		23/82	
**Age groups**	1432		
21 to 30 years		86	6.0
31 to 40 years		178	12.4
41 to 50 years		190	13.3
51 to 60 years		386	27.0
61 to 70 years		380	26.5
71 to 80 years		186	13.0
81 to 90 years		26	1.8
**Sex**	1429		
Female		814	57.0
Male		613	42.9
Divers		2	0.1
**Federal state**	1491		
Baden-Württemberg		308	20.7
Berlin		277	18.6
North Rhine-Westphalia		270	18.1
Saxony-Anhalt		306	20.5
Schleswig Holstein		330	22.1
**Political self-positioning** (10-point scale)	1489		
**mean ± SD**		4.4±1.6	
Left [1, 2]		183	12.3
Moderately left [3, 4]		547	36.7
Centre [5, 6]		636	42.7
Moderately right [7, 8]		109	7.3
Right [9, 10]		14	0.9
**Educational level (based on school degrees)**	1431		
High		937	62.9
Middle		401	26.9
Low		73	4.9
Other		4	0.3
Currently enrolled		16	1.1
**Average monthly net income of household (€)**	1302		
< 3000		566	43.5
3 000 to 5 000		498	38.2
5 000 Euro and more		238	18.3
**Physician visits within the last 12 months**	1478		
None		109	7.4
1 to 5		1066	72.1
> 5		303	20.5
Attitudes on climate change *	1491		
Alarmed		791	53.1
Concerned		457	30.7
Cautious		178	11.9
Disengaged		17	1.1
Doubtful		31	2.1
Dismissive		17	1.1

* According to Six Americas Short Survey [38]

### Attitudes towards climate change and prior participation in CSHC

Regarding their SASSY type, most participants were *alarmed* (*n* = 791, 53.1%), *concerned* (*n* = 457, 30.7%) or *cautious* (11.9%). Only 4.3% of the sample fell within one of the other three types combined (*disengaged, doubtful, dismissive*); see Table 3. Of all participants, 130 (8.7%) stated that CC and health topics had been part of their consultation with physicians at least once before. When asked about specific topics related to CSHC, 595 participants (39.9%) stated that their physicians had addressed at least one of these topics in the past (see Table 3).

**Table 3:** Climate-sensitive health counselling-related topics experienced by a subsample reporting at least one topic (*n* = 595)

Topics related to climate-sensitive health counselling	*n*	%
Questionnaires with at least one selected topic-related answer option for this item*:	**595**	**100.0%**
Spending time in nature to improve mental health	418	70.3
Healthy and climate-friendly mobility	150	25.2
Healthy and climate-friendly nutrition	121	20.3
Connection between CC and allergies	120	20.1
Connection between CC and infectious diseases	85	14.3
Health risks from heat waves due to CC	64	10.8
Connection between CC and the physical consequences of extreme weather events	47	7.9
Impairment of mental health due to concerns about CC	42	7.1
Impairment of mental health due to extreme weather events as a result of CC	41	6.9

* Multiple responses possible

### Preferred information channels for climate change and health topics

The largest proportion of respondents (74.4%) approved of flyers or brochures displayed in the physician’s office as an information channel. A total of 55.8% were in favour of personal consultation with a physician. Information on the practice’s website or posters in the practice rooms was supported by close to one-third of participants (34.3% and 32.5% respectively). For more details, see Supplement Table 2.1.

### General and content preferences for CSHC

Regarding general CSHC preference (Section F in Table 1), almost half of the participants (46.7%) (rather) wanted “*topics of climate change and health to be part of the consultation”* with their physician, 19.3% were indifferent, and one third (33.9%) (rather) did not want this (Table 4, first row). Regarding content, 62.3% wanted to be advised about how to protect their health from CC impacts, 53.1% wanted to be informed about CC impacts on their health, 48% wanted to talk about how to lead a healthy and climate-friendly lifestyle, 20.9% about fears and concerns regarding the impacts of CC and 9.4% about how to engage in climate action (see Table 4).

**Table 4:** Topic preferences for CSHC

				Percentage of respondents					*n*
		Mean*	SD	No, not at all	Rather no	Doesn’t matter	Rather yes	Yes, very much	
F. General CSHC preference		3.2	1.2	6.1	27.8	19.3	31.4	15.4	1491
D1. Impacts of climate change on health	a) In general	3.4	1.2	5.2	25.6	16.0	34.5	18.6	1491
	b) Heat waves	3.6	1.1	2.6	19.8	17.0	39.7	20.9	1485
	c) Pollen season/allergies	3.7	1.1	2.0	17.3	16.3	40.0	24.4	1481
	d) Infectious diseases	3.9	1.0	2.0	11.3	9.2	48.8	28.7	1483
D2. Talking about…	a) climate anxiety	2.4	1.1	14.1	48.6	16.4	16.7	4.2	1487
	b) climate action	2.1	0.9	23.3	58.4	8.9	7.2	2.2	1488
D3. Adaptation measures for climate change-related health issues	d) Infectious diseases	3.9	1.0	1.5	11.9	11.0	46.2	29.4	1486
	b) Heat waves	3.8	1.1	1.5	16.3	11.8	42.6	27.8	1487
	c) Pollen season/allergies	3.7	1.1	1.8	16.1	18.7	39.1	24.4	1482
	d) Infectious diseases	3.9	1.0	1.5	11.9	11.0	46.2	29.4	1486
D4. Links between climate change and…	a) personal health	3.7	1.1	3.6	17.4	13.0	41.4	24.6	1486
	b) population health	2.9	1.1	8.6	38.5	19.7	25.4	7.8	1483
D5. Climate-friendly lifestyle changes	a) In general	3.2	1.2	5.5	28.5	18.0	34.5	13.5	1491
	b) Healthy and climate-friendly nutrition	3.4	1.2	4.4	25.9	14.5	38.7	16.5	1485
	c) Healthy and climate-friendly mobility	2.8	1.1	8.6	39.6	19.7	23.0	9.1	1481
	d) Mental health	3.3	1.2	4.4	25.6	18.8	34.2	17.0	1484

* Mean and standard deviation of 5-point Likert scale (1: *No, not at all* to 5: *Yes, very much*)

The subitems for the topics of CC impacts (D1b-d), adaptation measures (D3b-d) and healthy and climate-friendly lifestyles (D5b-d) tended to be rated more positively than the general items (D1a, D3a, D5a), as reflected in Table 4. With regard to specific topics on CC impacts and adaptation, participants showed the greatest preference to be informed about impacts on infectious diseases (D1.d; 77.5%), to be advised on how to protect their health from CC-related changes in the spread of infectious diseases (D3.d; 75.6%) and on how to protect their health and that of their next-of-kin on hot days and in heat waves (D3.b; 70.4%) (see Table 4). With regard to lifestyles, more than half of the participants wanted to talk about how to eat healthier and more climate-friendly (D5.b: 55.2%) and how to improve their mental wellbeing by spending time in nature (D5.d: 51.7%). Only 32.1% wanted to talk about healthier and more climate-friendly mobility options (D5.c). The respondents showed a greater preference for talking about the links between CC and their personal health (66.1%) than for talking about the links to population health (33.2%) (see Table 4).

### Associations between sociodemographic and attitudinal characteristics and CSHC preference

Univariate regressions showed some significant correlations between the independent variables and dependent variable. In the multiple regression model, age, SASSY type and political self-positioning were significantly associated with general preference for CSHC (see Table 5). Being younger than 50 years of age increased the preference for CSHC. Individuals who were *alarmed* about CC (versus *concerned*/*cautious*/*disengaged*/*doubtful*/*dismissive*) showed higher preference for CSHC. The further participants positioned themselves to the political right, the lower their CSHC preference was. The full model including age, sex, income, education, SASSY type, political self-positioning as independent variables explained 27.6% of the variance in participants’ CSHC preference (F (19, 1276) = 25.56, *p* < 0.001, *R²* = 0.276). With VIF values close to 1, no relevant signs of multicollinearity were found (see Supplementary Table 2.2. and 2.3.).

**Table 5:** Multiple linear regression model of individual characteristics (independent variable) and preference for CSHC (dependent variable)

Independent variables*		Models					
		Model 1		Model 2		Model 3	
		Unstandardized coefficients (95% confidence intervals)					
Sex	Male vs. Female	-0.03	(-0.17; 0.10)	-0.05	(-0.18; 0.09)	0.11	(-0.01; 0.23)
Age (grouped)	21 - 30y vs. 51 - 60y	0.5	(0.21; 0.79)	0.49	(0.20; 0.79)	0.26	(0.00; 0.52)
	31 - 40y vs. 51 - 60y	0.31	(0.10; 0.53)	0.31	(0.08; 0.53)	0.23	(0.04; 0.42)
	41 - 50y vs. 51 - 60y	-0.08	(-0.29; 0.13)	-0.09	(-0.30; 0.13)	0	(-0.18; 0.19)
	61 - 70y vs. 51 - 60y	0.11	(-0.06; 0.29)	0.11	(-0.07; 0.29)	0.03	(-0.13; 0.18)
	71 - 80y vs. 51 - 60y	0.03	(-0.19; 0.25)	0.03	(-0.19; 0.25)	-0.06	(-0.26; 0.13)
	81 - 90y vs. 51 - 60y	0.01	(-0.48; 0.49)	-0.01	(-0.50; 0.48)	-0.07	(-0.49; 0.35)
Income (grouped)	Middle vs. Low			0.04	(-0.11; 0.18)	-0.06	(-0.19; 0.06)
	High vs. Low			0.03	(-0.16; 0.22)	-0.07	(-0.23; 0.10)
Education (grouped)	Low vs. High			0.1	(-0.20; 0.41)	0.26	(0.00; 0.53)
	Middle vs. High			-0.07	(-0.22; 0.08)	0.11	(-0.02; 0.24)
	Currently pupil vs. High			-0.11	(-1.27; 1.05)	0.32	(-0.69; 1.32)
	Other vs. High			0.21	(-0.38; 0.79)	0.17	(-0.34; 0.67)
SASSY type	Concerned vs. Alarmed					-0.66	(-0.79; -0.53)
	Cautious vs. Alarmed					-1.39	(-1.58; -1.20)
	Disengaged vs. Alarmed					-1.41	(-1.99; -0.82)
	Doubtful vs. Alarmed					-1.96	(-2.36; -1.57)
	Dismissive vs. Alarmed					-2.24	(-2.77; -1.71)
Political self-positioning	Political self-positioning**					-0.06	(-0.10; -0.03)

*95% confidence intervals given in brackets; R^2^ = 0.02 for Model 1, △R^2^ = 0.002 for Model 2 (*p* = 0.87), △R^2^ = 0.26 for Model 3 (*p* < 0.001); *n* = 1296

** continuous, per point on the scale of 10, from 1 (left) to 10 (right)

### Sensitivity analysis

To validate the outcome measure “general CSHC preference” (Section F in Table 1), we built a composite measure consisting of all the items on topic preferences regarding CSHC, namely D1b/D1c/D1d, D3b/D3c/D3d, D5b/D5c/D5d, D2a/b and D4a/b (see Tables 1 and 4) which had an internal validity of Cronbach’s α = 0.94. As the mean of this composite measure (3.30 ± 0.83) and that of the primary outcome variable form section F (3.22 ± 1.18) were similar, we decided to retain the latter for the final regression model. Based on the structure of the questionnaire, we conclude that item F best reflects the participants’ general view of CSHC and thus serves as appropriate outcome to our analyses: the item asks about general preference for CSHC at the very end of the questionnaire after the participants have learnt about concepts and possible topics of such a consultation.

## Discussion

We assessed preferences for and previous participation in CSHC among German adults in the HeReCa panel. A total of 8.7% of the participants reported having participated in CSHC previously and 39.9% had previously discussed at least one CSHC-related topic with physicians before. Almost half of the sample wanted CC and health topics to be part of a medical consultation, whereas one third did not. Regarding topic preferences, more than two thirds wanted information about the health impact of infectious diseases and advice on adaptation measures regarding infectious diseases and heat. Half of the participants wanted to talk about climate-friendly and healthy lifestyles. Multivariable regression of the association between socioeconomic and attitudinal variables and general CSHC preference indicated that younger participants, those with an *alarmed* CC attitude and those politically oriented to the left (political self-positioning on a 10-point scale from left to right), had greater preference for CSHC.

In this sample, 39.9% had experienced at least one of the suggested topics of CSHC in a consultation, particularly *“Spending time in nature to improve mental health”*. CSHC is a broad concept and spending time in nature can be part of CSHC when physicians intend to not only improve their patients’ mental health but also strengthen their connection to nature to possibly increase their pro-environmental behaviour [25, 39]. However, only 8.7% of participants reported having participated in CSHC as a noticeable concept. This lies within the range of previous findings from the US and Germany indicating that between 4% and 15% of the respondents had participated in such counselling formats [26–28]. While more than half of the participants welcomed CSHC, approximately one third rejected it. According to a qualitative study among patients, possible reasons could be disinterest in CC, feelings of guilt and shame, or concern that there will not be enough time to discuss the patient’s health problems [18].

### Content preferences

There was a clear preference for CSHC related to respondents’ personal health compared to CSHC related to population health. This finding is in line with other research from clinical settings in which physicians indicated that CSHC and advice on lifestyle interventions should be implemented only when they can be well connected to a patient’s existing health problems [29, 40]. Griesel et al. also found that a link to one’s personal health contributed to patients’ acceptance of CSHC [18].

We found that regarding general content, the greatest preference was for advice on adaptation measures (framed as health protection). This may reflect people’s basic need to protect their health by learning how to adapt to potential hazards [41]. In terms of specific topics related to CC impacts and adaptation, it is noticeable that the highest preference was for infectious diseases compared to allergies and heat. We did not find other studies assessing information interest in CSHC. However, the PACE study revealed that almost three quarters of a representative German population sample perceived CC impacts on heat-related (73%) and infectious diseases (72%) as severe, while this was only the case for 49% of the population with regard to allergies [42]. Furthermore, Schmuker et al. reported that 72% of people in Germany felt well informed about the heat health impacts of CC and less well informed about infectious disease impacts (65% regarding vector-borne diseases, 50% food-borne and 47% water-borne) or allergies (53%) [43]. This might suggest that interest in counselling on infectious diseases might be high, as perceived severity is high and perceived personal knowledge is low for infectious diseases. However, this can only be considered as a hypothesis and warrants further research.

With regard to lifestyle topics, half of our participants were interested in receiving advice on climate-friendly nutrition or the connection between mental health and CC, and only one third were interested in receiving advice on climate-friendly mobility. According to the CC literature, people are more willing to change their dietary habits than their mobility habits when asked to reduce their greenhouse gas emissions [44].

Compared to information on CC impacts, CC adaptation and lifestyles, a smaller share of participants (20.9%) wanted to talk about fears or concerns related to CC. Climate anxiety levels in Germany are reported to be low [45, 46]. For instance, König and Hajek found a mean Climate Anxiety Score of 2.0 on a scale from 1 to 7 [46]. This suggests that many people do not have severe CC worries and, consequently, that there is no need to talk about them. Nevertheless, physicians should be ready to give room to talk about feelings related to CC with their patients [25], as one fifth of participants wanted to talk about worries, indicating that they had experienced them.

### Individual characteristics associated with CSHC preference

Income and education were not associated with CSHC preferences, which corresponds to findings from a study by the German Federal Ministry of the Environment showing that different types of environmental awareness cannot be clearly assigned to social strata based on income or education [47]. Engels et al. and Horndsey et al. found that value-based ideologies and political orientations have much greater explanatory value for climate scepticism and belief in CC than education and income [48, 49].

In most European countries, it was found that political self-positioning further to the right is correlated with less worrying about CC [50]. Therefore, it seems reasonable that political orientation further to the right was negatively associated with preference for CSHC in our sample.

Additionally, being alarmed about CC had the strongest positive correlation with wanting to receive CSHC compared to being concerned, cautious, disengaged, doubtful or dismissive. The SASSY score used for this classification includes one item on how much one thinks CC will harm oneself personally [34]. According to protection motivation theory, the perceived severity and perceived personal vulnerability of a (health) risk contribute to the intention to adopt a protective (health) behaviour [51]. It was also shown that experiencing extreme weather events was a strong predictor of information seeking behaviour related to CC adaptation measures [52]. This might explain why greater CC risk perception is positively correlated with greater preference for CSHC, which provides information on climate-related health risks and related adaptation strategies.

Furthermore, younger age was associated with greater CSHC preference in our sample, even after correcting for attitudes towards CC. This result is in line with other findings showing that younger generations have a greater emotional engagement with CC, see a more urgent need for change in climate protection and show less skepticism regarding CC than older age groups [53–56].

### Strengths and limitations

To the best of our knowledge, this is the first survey dedicated to specifically investigating participation in and preferences for CSHC in the general population, both in Germany and internationally. While there was no existing tool for measuring the concept of CSHC, we see it as a strength of this study that we could draw on the experience of a scoping review and qualitative studies about CSHC conducted within our research team to build this questionnaire [18, 25, 37]. Furthermore, we were able to draw on the existing HeReCa panel, constructed for general health surveys rather than CC and health research, which was one way to reduce selection bias regarding the participation of people with a general affinity for CC topics. Nevertheless, with a response rate of 47.1% (similar to other HeReCa surveys [57, 58]), possible selection bias remains a limitation of this study. Moreover, neither the HeReCa panel nor the investigated sample is representative of the German population. Although political self-positioning corresponds relatively well to the German average (mean 4.4 ± 1.6 vs. 4.9 ± 1.8 in ALLBUS data 2021 [59]), the study population is more female-dominated, older and has better school education levels than the German adult population [60–62]. Another limitation is the lack of validation of the 4-item SASSY characterizing CC attitudes in the German population. The long version with 36 items has recently been adapted to Germany [63], yet it was not feasible to use this long version for our study. The SASSY was a useful tool for segmenting our sample into groups differing in their CC attitude to measure associations with CSHC preference. However, the groups *disengaged* (*n* = 17), *doubtful* (*n* = 31) and *dismissive* (*n* = 17) were very small in our sample, so the results for these groups from the regression model need to be interpreted with care.

### Implications for practice

The preference of almost half of the participants to receive CHSH, and even greater preference to be informed or advised on specific topics, underlines that physicians should be trained on CC and health issues [64], as well as in dealing with different preferences in this respect. For many physicians, not feeling prepared for CSHC is the greatest barrier to its implementation [26, 65, 66]. Topicwise, a focus on the links between CC and personal health issues and the use of specific information about impacts and adaptation measures seems suitable for performing CSHC. Information material was seen as another appropriate information channel for CC and health issues and might be a less intrusive way to communicate related issues to patients, which could also be combined with individual counselling.

This study revealed associations between political self-positioning, CC attitudes and CSHC preference. As CSHC should be delivered in a patient-centred manner and consider the (environmental) values of patients [25, 67], our findings underscore the need for physicians not to provide CSHC uniformly to all patients but to tailor it to patients’ values and needs. To do so, it can be helpful to know patients’ CC-related attitudes or to explore these attitudes. These may already be known to primary care physicians due to their long-standing relationship with patients. In addition, it can be legitimate to elicit patients’ perspectives regarding CC to individualize CSHC. Identifying patients’ values or motivations is a recommended first step in several health counselling techniques such as patient-centred communication or motivational interviewing [68, 69]. One example of such tailoring might be that recommendations on the healthfulness of walking small distances instead of using the car could be given to any patient but that the climate cobenefit is only explicitly mentioned as one more motivator for those patients who consider CC mitigation to be relevant to them.

## Conclusions

Despite the relevance of CSHC for disease prevention and health promotion in a rapidly changing climate, participants reported little previous participation in such counselling. Almost half of the respondents would value CSHC by physicians, and up to three quarters were interested in learning about specific content related to CC and health. Therefore, physicians and other health professionals should be trained in patient-centred CSHC to be able to respond to this emerging health need. Further studies should assess the effects of CSHC on patients’ health and climate-related knowledge, attitudes and behaviours to establish its function in disease prevention and health promotion in the health system.

### Electronic supplementary material

Below is the link to the electronic supplementary material.

Supplementary Material 1

## Data Availability

More detailed analyses are provided in the supplementary material. The datasets used and/or analyzed during the current study are available from the corresponding author on reasonable request.

## Abbreviations

CC: Climate change
CSHC: Climate-sensitive health counselling
