# Supplementary material for: Climate-sensitive health counselling in Germany: a cross-sectional study about previous participation and preferences in the general public
Source: BMC Public Health. 2024 Jun 6;24:1519. doi: 10.1186/s12889-024-18998-6 (PMC11155184; doi:10.1186/s12889-024-18998-6)
Supplement: Supplementary file 1 — Supplementary Material 1 [file 12889_2024_18998_MOESM1_ESM.docx]

**Supplementary Material**

**Article title:** Climate-sensitive health counselling in Germany: a cross-sectional study about previous participation and preferences in the general public

**Table of Contents**

1. FIGURES
   1. Flow diagram of the sample’s construction and recruitment process
   2. SASSY types regarding participants’ attitudes on climate change
   3. Readiness for climate-friendly behaviour (percentage figures)
2. TABLES
   1. Preferred information channels for information on the relationship between climate change and health
   2. Linear regression model of individual characteristics and preference for climate-sensitive health counselling (dependent variable)
   3. Linear regression model of individual characteristics (federal states as additional independent variables) and preference for climate-sensitive health counselling (dependent variable)
   4. Summary of sociodemographic and attitudinal characteristics including those of participants with missing data
3. QUESTIONNAIRES
   1. German original version
   2. English translation
4. **FIGURES**

**Figure 1.1.: Flow diagram of the sample’s construction and recruitment process**

*
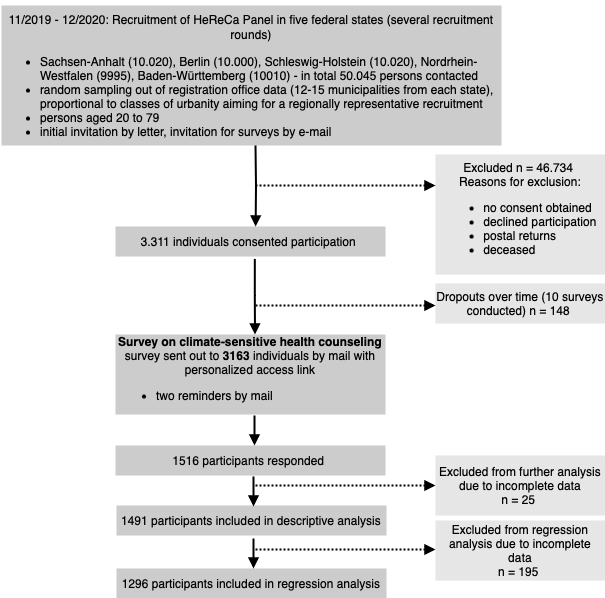
*

**Figure 1.2.: SASSY types regarding participants’ attitudes on climate change***

* based on a four-item survey tool (Chryst et al., 2018) segmenting survey respondents into six types, percentages of total sample, n = 1491


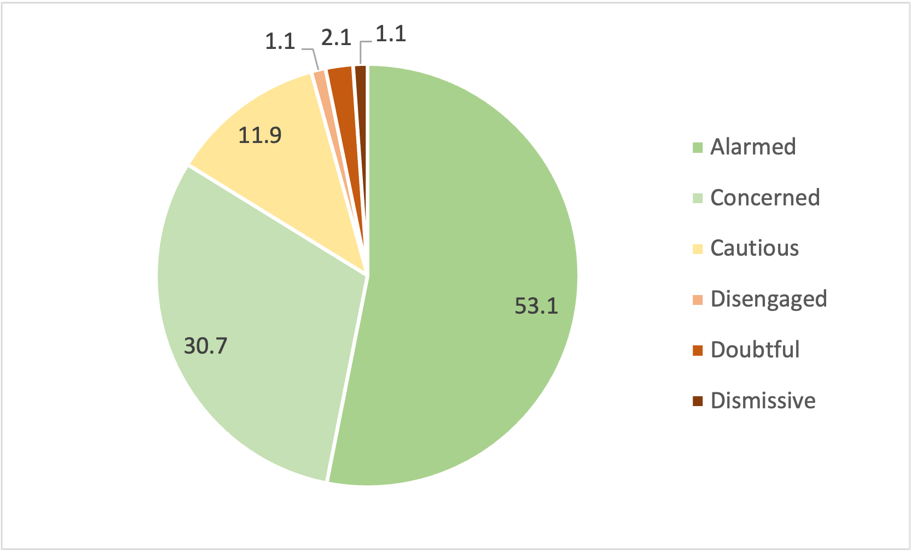


**Figure 1.3.: Readiness for climate-friendly behaviour (percentage figures)**


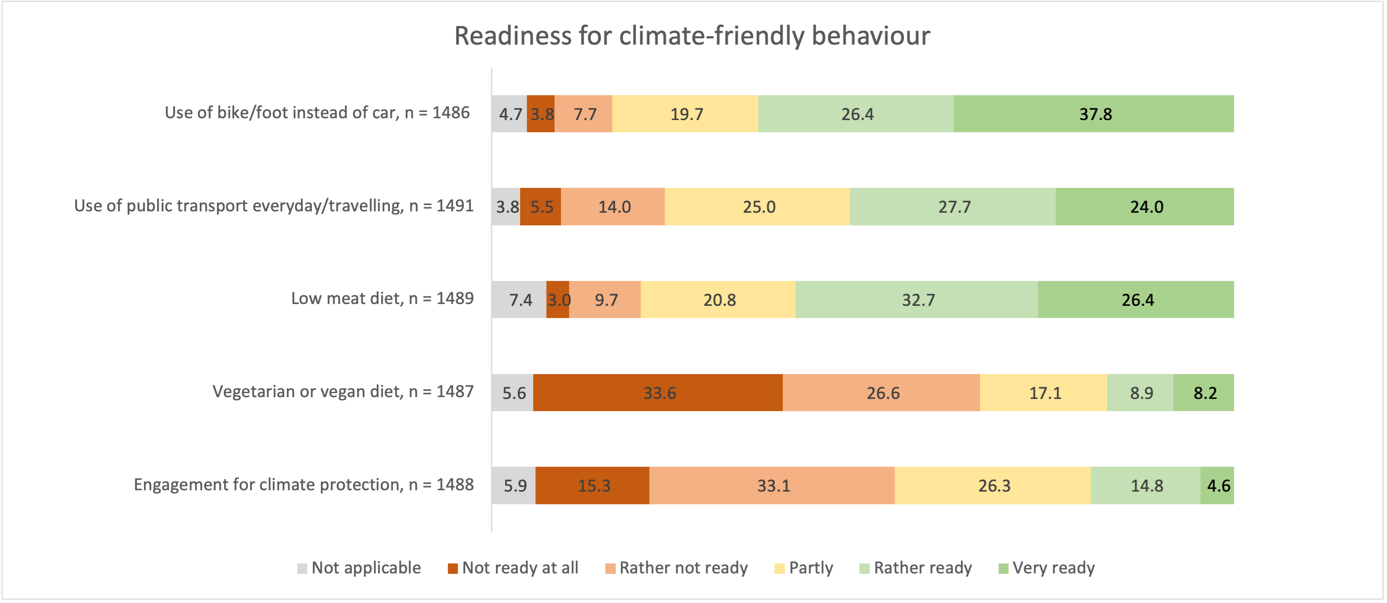


1. **TABLES**

**Table 2.1.: Preferred information channels for information on the relationship between climate change and health***

| Information channel | **n** |
| --- | --- |
| flyers/booklets in the physician’s office | 948 |
| personal conversation | 712 |
| internet presence of the practice | 437 |
| posters in the practice | 414 |
| newsletter of the practice | 270 |
| social media presence of the practice | 201 |
| none of the above information channels are suitable | 216 |

* Multiple responses possible, n = 1275

**Table 2.2.: Linear regression model of individual characteristics and preference for climate-sensitive health counselling (dependent variable)***

|  | Independent variable | | b | SE | 95% CI | | p | VIF |
| --- | --- | --- | --- | --- | --- | --- | --- | --- |
|  |  |  | |  | lower limit | upper  limit |  |  |
| **Model 1** |  | |  |  |  |  |  |  |
| **Sex**  **Age**  (grouped) | Constant  male vs. female  21 - 30y vs. 51 - 60y  31 - 40y vs. 51 - 60y  41 - 50y vs. 51 - 60y  61 - 70y vs. 51 - 60y  71 - 80y vs. 51 - 60y  81 - 90y vs. 51 - 60y | | 3.14  -0.03  **0.50**  **0.31**  -0.08  0.11  0.03  0.01 | 0.07  0.07  **0.15**  **0.11**  0.11  0.09  0.11  0.25 | 3.01  -0.17  **0.21**  **0.10**  -0.29  -0.06  -0.19  -0.48 | 3.28  0.10  **0.79**  **0.53**  0.13  0.29  0.25  0.49 | <0.01  0.61  **<0.01**  **<0.01**  0.46  0.21  0.79  0.98 | 1.05  **1.16**  **1.30**  1.30  1.46  1.31  1.06 |
| **Model 2** |  | |  |  |  |  |  |  |
| **Sex**  **Age**  (grouped)  **Income**  (grouped)  **Education**  (grouped) | Constant  male vs. female  21 - 30y vs. 51 - 60y  31 - 40y vs. 51 - 60y  41 - 50y vs. 51 - 60y  61 - 70y vs. 51 - 60y  71 - 80y vs. 51 - 60y  81 - 90y vs. 51 - 60y  middle vs. low  high vs. low  low vs. high  middle vs. high  currently pupil vs. high  other vs. high | | 3.14  -0.05  **0.49**  **0.31**  -0.09  0.11  0.03  -0.01  0.04  0.03  0.10  -0.07  -0.11  0.21 | 0.09  0.07  **0.15**  **0.11**  0.11  0.09  0.11  0.25  0.07  0.10  0.16  0.08  0.59  0.30 | 2.97  -0.18  **0.20**  **0.08**  -0.30  -0.07  -0.19  -0.50  -0.11  -0.16  -0.20  -0.22  -1.27  -0.38 | 3.32  0.09  **0.79**  **0.53**  0.13  0.29  0.25  0.48  0.18  0.22  0.41  0.08  1.05  0.79 | <0.01  0.51  **<0.01**  **<0.01**  0.43  0.23  0.79  0.98  0.63  0.75  0.52  0.37  0.86  0.49 | 1.07  **1.24**  **1.34**  1.32  1.49  1.33  1.06  1.25  1.31  1.07  1.14  1.01  1.01 |
| **Model 3** |  | |  |  |  |  |  |  |
| **Sex**  **Age**  (grouped)  **Income**  (grouped)  **Education**  (grouped)  **Attitudinal type**  **Political self-positioning** | Constant  male vs. female  21 - 30y vs. 51 - 60y  31 - 40y vs. 51 - 60y  41 - 50y vs. 51 - 60y  61 - 70y vs. 51 - 60y  71 - 80y vs. 51 - 60y  81 - 90y vs. 51 - 60y  middle vs. low  high vs. low  low vs. high  middle vs. high  currently pupil vs. high  other vs. high  concerned vs. alarmed  cautious vs. alarmed  disengaged vs. alarmed  doubtful vs. alarmed  dismissive vs. alarmed  Political self-positioning** | | 3.85  0.11  **0.26**  **0.23**  0.00  0.03  -0.06  -0.07  -0.06  -0.07  0.26  0.11  0.32  0.17  **-0.66**  **-1.39**  **-1.41**  **-1.96**  **-2.24**  **-0.06** | 0.11  0.06  **0.13**  **0.10**  0.09  0.08  0.10  0.22  0.07  0.08  0.14  0.07  0.51  0.26  **0.07**  **0.10**  **0.30**  **0.20**  **0.27**  **0.02** | 3.64  -0.01  **0.00**  **0.04**  -0.18  -0.13  -0.26  -0.49  -0.19  -0.23  0.00  -0.02  -0.69  -0.34  **-0.79**  **-1.58**  **-1.99**  **-2.36**  **-2.77**  **-0.10** | 4.06  0.23  **0.52**  **0.42**  0.19  0.18  0.13  0.35  0.06  0.10  0.53  0.24  1.32  0.67  **-0.53**  **-1.20**  **-0.82**  **-1.57**  **-1.71**  **-0.03** | 0.00  0.07  **<0.05**  **<0.05**  0.97  0.71  0.51  0.75  0.33  0.42  0.05  0.10  0.53  0.51  **<0.01**  **<0.01**  **<0.01**  **<0.01**  **<0.01**  **<0.01** | 1.09  **1.25**  **1.35**  1.33  1.51  1.34  1.06  1.28  1.33  1.08  1.17  1.02  1.02  **1.14**  **1.28**  **1.02**  **1.08**  **1.06**  **1.22** |

*95% confidence intervals given; R^2^= 0.02 for Model 1, △R^2^ = 0.00 for Model 2 (p = 0.87), △R^2^ = 0.26 for Model 3 (p < 0.001); sex, age, income, education, federal states and attitudinal types are dummycoded, n = 1296, significant values in bold

** scale of 10, from 1 (left) to 10 (right)

**Table 2.3.: Linear regression model of individual characteristics (including federal states) and preference for climate-sensitive health counselling (dependent variable)***

|  | Independent variable | b | SE | 95% CI | | p | VIF |
| --- | --- | --- | --- | --- | --- | --- | --- |
|  |  |  |  | lower limit | upper limit |  |  |
| **Step 1** |  |  |  |  |  |  |  |
| **Sex**  **Age**  (grouped) | Constant  male vs. female  21 - 30y vs. 51 - 60y  31 - 40y vs. 51 - 60y  41 - 50y vs. 51 - 60y  61 - 70y vs. 51 - 60y  71 - 80y vs. 51 - 60y  81 - 90y vs. 51 – 60y | 3.14  -0.03  **0.50**  **0.31**  -0.08  0.11  0.03  0.01 | 0.07  0.07  **0.15**  **0.11**  0.11  0.09  0.11  0.25 | 3.01  -0.17  **0.21**  **0.10**  -0.29  -0.06  -0.19  -0.48 | 3.28  0.10  **0.79**  **0.53**  0.13  0.29  0.25  0.49 | 0.00  0.61  **<0.01**  **<0.01**  0.46  0.21  0.79  0.98 | 1.05  **1.16**  **1.30**  1.30  1.46  1.31  1.09 |
| **Step 2** |  |  |  |  |  |  |  |
| **Sex**  **Age**  (grouped)  **Income**  (grouped)  **Education**  (grouped)  **Federal state** | Constant  male vs. female  21 - 30y vs. 51 - 60y  31 - 40y vs. 51 - 60y  41 - 50y vs. 51 - 60y  61 - 70y vs. 51 - 60y  71 - 80y vs. 51 - 60y  81 - 90y vs. 51 - 60y  middle vs. low  high vs. low  low vs. high  middle vs. high  currently pupil vs. high  other vs. high  Baden-Württemberg vs. Schleswig-Holstein  Berlin vs. Schleswig-Holstein (SH)  Nordrhein-Westfalen vs. SH  Sachsen-Anhalt vs. SH | 3.15  -0.04  **0.49**  **0.29**  -0.07  0.11  0.02  -0.02  0.03  0.01  0.08  -0.03  -0.04  0.18  -0.05  0.11  0.11  -0.19 | 0.11  0.07  **0.15**  **0.11**  0.11  0.09  0.11  0.25  0.08  0.10  0.16  0.08  0.59  0.30  0.10  0.10  0.10  0.10 | 2.93  -0.17  **0.19**  **0.07**  -0.28  -0.07  -0.20  -0.50  -0.12  -0.19  -0.23  -0.18  -1.20  -0.41  -0.25  -0.09  -0.10  -0.39 | 3.37  0.09  **0.79**  **0.51**  0.15  0.29  0.25  0.47  0.17  0.20  0.39  0.13  1.12  0.76  0.15  0.31  0.31  0.01 | 0.00  0.54  **<0.01**  **<0.01**  0.54  0.22  0.83  0.95  0.72  0.96  0.62  0.72  0.94  0.55  0.64  0.29  0.31  0.06 | 1.07  **1.25**  **1.35**  1.33  1.49  1.34  1.07  1.27  1.35  1.09  1.17  1.01  1.02  1.52  1.52  1.50  1.59 |
| **Step 3** |  |  |  |  |  |  |  |
| **Sex**  **Age**  (grouped)  **Income**  (grouped)  **Education**  (grouped)  **Federal state**  **Attitudinal type**  **Political selfpositioning** | Constant  male vs. female  21 - 30y vs. 51 - 60y  31 - 40y vs. 51 - 60y  41 - 50y vs. 51 - 60y  61 - 70y vs. 51 - 60y  71 - 80y vs. 51 - 60y  81 - 90y vs. 51 - 60y  middle vs. low  high vs. low  low vs. high  middle vs. high  currently pupil vs. high  other vs. high  Baden-Württemberg vs. Schleswig Holstein  Berlin vs. Schleswig Holstein (SH)  North Rhine-Westfalen vs. SH  Saxony-Anhalt vs. SH  concerned vs. alarmed  cautious vs. alarmed  disengaged vs. alarmed  doubtful vs. alarmed  dismissive vs. alarmed  Political self-positioning** | 3.81  0.11  **0.26**  **0.23**  0.01  0.03  -0.07  -0.07  -0.06  -0.07  0.26  0.13  0.35  0.15  -0.02  0.12  0.10  -0.04  **-0.65**  **-1.38**  **-1.39**  **-1.96**  **-2.23**  **-0.06** | 0.12  0.06  **0.13**  **0.10**  0.09  0.08  0.10  0.22  0.07  0.09  0.14  0.07  0.51  0.26  0.09  0.09  0.09  0.09  **0.07**  **0.10**  **0.30**  **0.20**  **0.27**  **0.02** | 3.58  -0.01  **0.00**  **0.04**  -0.17  -0.13  -0.26  -0.49  -0.19  -0.24  -0.01  0.00  -0.66  -0.35  -0.19  -0.06  -0.08  -0.21  **-0.78**  **-1.57**  **-1.98**  **-2.35**  **-2.76**  **-0.10** | 4.05  0.23  **0.52**  **0.42**  0.20  0.18  0.13  0.36  0.07  0.10  0.53  0.27  1.35  0.66  0.15  0.29  0.27  0.14  **-0.53**  **-1.19**  **-0.81**  **-1.56**  **-1.70**  **-0.03** | 0.00  0.06  **<0.05**  **<0.05**  0.89  0.71  0.50  0.76  0.36  0.41  0.05  0.06  0.50  0.56  0.82  0.19  0.27  0.68  **<0.01**  **<0.01**  **<0.01**  **<0.01**  **<0.01**  **<0.01** | 1.10  **1.26**  **1.35**  1.33  1.51  1.34  1.07  1.29  1.36  1.10  1.20  1.02  1.02  1.52  1.53  1.51  1.61  **1.15**  **1.30**  **1.03**  **1.09**  **1.06**  **1.24** |

*95% confidence intervals given; R^2^= 0.017 for Step 1, △R^2^ = 0.015 for Step 2 (p = 0.21), △R^2^ = 0.251 for Step 3 (p < 0.001); sex, age, income, education, federal states and attitudinal types are dummycoded; n = 1296, significant values in bold

**scale of 10, from 1 (left) to 10 (right)

**Table 2.4.: Summary of sociodemographic and attitudinal characteristics including those of participants with missing data (fourth row)**

|  | **n (total)** | **n** | **%** | **% Missings (n = 195)** |
| --- | --- | --- | --- | --- |
| **Age (years) mean ± SD**  **Age min/max**  **Age groups**  21 to 30 years  31 to 40 years  41 to 50 years  51 to 60 years  61 to 70 years  71 to 80 years  81 to 90 years | 1432 | 55.6 ±14.2  23/82  86  178  190  386  380  186  26 | 6.0  12.4  13.3  27.0  26.5  13.0  1.8 | 58±12.9  25/82  (n = 136)  5.1  8.1  9.6  28.7  31.6  15.4  1.5 |
| **Sex**  female  male  divers | 1429 | 814  613  2 | 57.0  42.9  0.1 | (n =133)  62.4  36.1  1.5 |
| **Federal state**  Baden-Württemberg  Berlin  North Rhine-Westphalia  Saxony-Anhalt  Schleswig Holstein | 1491 | 308  277  270  306  330 | 20.7  18.6  18.1  20.5  22.1 | (n = 195)  29.7  17.9  17.4  16.4  18.5 |
| **Political self-positioning** (10-point scale)  **mean ± SD**  left (1,2)  moderately left (3, 4)  centre (5, 6)  moderately right (7, 8)  right (9, 10) | 1489 | 4.4±1.6  183  547  636  109  14 | 12.3  36.7  42.7  7.3  0.9 | (n = 193)  4.42±1.6  11.9  36.3  44.0  7.3  0.5 |
| **Educational level (based on school degrees)**  high  middle  low  other  currently enrolled | 1431 | 937  401  73  4  16 | 62.9  26.9  4.9  0.3  1.1 | (n= 135)  64.4  28.9  6.7  0  0 |
| **Average monthly net income of household (€)**  < 3000  3 000 to 5 000  5 000 Euro and more | 1302 | 566  498  238 | 43.5  38.2  18.3 | (n = 6)  66.7  16.7  16.7 |
| **Physician visits within the last 12 months**  none  1 to 5  > 5 | 1478 | 109  1066  303 | 7.4  72.1  20.5 | (n = 195)  7.7  71.8  20.5 |
| **Attitudes on climate change ***  alarmed  concerned  cautious  disengaged  doubtful  dismissive | 1491 | 791  457  178  17  31  17 | 53.1  30.7  11.9  1.1  2.1  1.1 | (n = 195)  55.4  29.2  10.3  2.6  1.5  1.0 |

* According to Six Americas Short Survey (30)

1. **QUESTIONNAIRES**

**Questionnaire 3.1.: German original version**

(*markiert verpflichtende Items)

*Einleitung:*

*Es gibt verschiedene Zusammenhänge zwischen Klimawandel und Gesundheit. Wir wollen herausfinden, ob diese auch im Arzt-Patienten-Gespräch eine Rolle spielen sollten. Dazu interessiert uns Ihre Meinung.*

*Nun folgen einige Fragen zu Ihrer Haltung bezüglich des Klimawandels.*

| **Aa) Wie wichtig ist Ihnen das Thema Klimawandel persönlich?*** | - Extrem wichtig - Sehr wichtig - Etwas wichtig - Nicht sehr wichtig - Überhaupt nicht wichtig |
| --- | --- |
| **Ab) Wie besorgt sind Sie in Bezug auf den Klimawandel?*** | - Sehr besorgt - Etwas besorgt - Nicht sehr besorgt - Überhaupt nicht besorgt |
| **Ac) Wie sehr denken Sie, dass der Klimawandel Ihnen selbst schaden wird?*** | - Sehr - Mäßig - Nur ein bisschen - Überhaupt nicht - Ich weiß es nicht |
| **Ad) Wie sehr denken Sie, dass der Klimawandel zukünftigen Generationen von Menschen schaden wird?*** | - Sehr - Mäßig - Nur ein bisschen - Überhaupt nicht - Ich weiß es nicht |

*Die folgenden Fragen handeln davon, inwiefern Sie es für wichtig halten, sich klimafreundlich zu verhalten.*

| **Ba) Sind Sie im Alltag bemüht, Ihren Treibhausgasausstoß zu verringern (d.h. Ihre klimaschädlichen Emissionen)?** | - Ja, sehr - Eher ja - Teils teils - Eher nein - Keinesfalls |
| --- | --- |
| **Wie sehr sind Sie bereit…?** |  |
| **Bb) … im Alltag ein Rad/E-Bike zu nutzen anstatt eines kraftstoffverbrauchenden Fahrzeugs?** | - Sehr bereit - Eher bereit - Teils teils - Eher nicht bereit - Gar nicht bereit - Nicht zutreffend |
| **Bc) … klimafreundliche öffentliche Verkehrsmittel (z.B. Bus, Bahn) im Alltag und für Reisen zu nutzen – statt des Autos oder Flugzeugs?** | - Sehr bereit - Eher bereit - Teils teils - Eher nicht bereit - Gar nicht bereit - Nicht zutreffend |
| **Bd) … sich fleischarm zu ernähren? (d.h. wenige Fleisch- und Wurstwaren) Wenn Sie sich schon rein vegetarisch ernähren, dann wählen Sie „Nicht zutreffend“ aus.** | - Sehr bereit - Eher bereit - Teils teils - Eher nicht bereit - Gar nicht bereit - Nicht zutreffend |
| **Be) … sich vollständig vegetarisch oder vegan zu ernähren? (d.h. Verzicht auf Fleisch- sowie Wurstwaren oder kompletter Verzicht auf tierische Produkte)** | - Sehr bereit - Eher bereit - Teils teils - Eher nicht bereit - Gar nicht bereit - Nicht zutreffend |
| **Bf) … sich ehrenamtlich, z.B. in einer Organisation oder einem Verein für Klimaschutz zu engagieren?** | - Sehr bereit - Eher bereit - Teils teils - Eher nicht bereit - Gar nicht bereit - Nicht zutreffend |

*Es folgen Fragen zu klimasensibler Gesundheitsberatung bei Ihrem Arzt oder Ihrer Ärztin. Klimasensible Gesundheitsberatung bedeutet, dass Themen zu Klimawandel und Gesundheit im ärztlichen Gespräch vorkommen.*

| **Ca) Waren solche Inhalte zu Klimawandel und Gesundheit Ihrer Meinung nach schon einmal Thema in einer Sprechstunde mit Ihrem Arzt/Ihrer Ärztin?*** | - Ja, oft - Ja, mindestens ein Mal - Nein - Weiß nicht |
| --- | --- |
| **Cb) Wählen Sie bitte aus, ob folgende Themen schon einmal von einem Arzt/einer Ärztin angesprochen wurden. Gerne können Sie auch “Keines dieser Themen“ ankreuzen oder das Freitextfeld nutzen, um etwas zu ergänzen.*** | - Gesundheitsrisiken durch Hitzewellen infolge des Klimawandels - Zusammenhang von Klimawandel und Allergien - Zusammenhang von Klimawandel und den körperlichen Folgen von Extremwetterereignissen - Beeinträchtigung der seelischen Gesundheit durch Extremwetterereignisse infolge des Klimawandels - Zusammenhang von Klimawandel und Infektionserkrankungen - Beeinträchtigung der seelischen Gesundheit durch Sorgen bezüglich des Klimawandels - Gesunde und klimafreundliche Ernährung - Gesunde und klimafreundliche Fortbewegung - Aufenthalt in der Natur zur Verbesserung des seelischen Wohlbefindens - Keines dieser Themen - Sonstiges: *(****Freitextfeld****)* |

*Der Klimawandel wirkt sich in Deutschland in unterschiedlicher Weise auf unsere Gesundheit aus. In den folgenden Fragen lernen Sie verschiedene Auswirkungen kennen, welche alle wissenschaftlich erforscht worden sind. Sie können nun Angaben dazu machen, welche Themen Sie im Rahmen einer klimasensibler Gesundheitsberatung interessieren würden. Berücksichtigen Sie dabei besonders, dass diese Themen in einem Gespräch mit Ihrem Arzt/Ihrer Ärztin vorkommen würden.*

| **D1a) Möchten Sie von Ihrem Arzt/Ihrer Ärztin über die Auswirkungen des Klimawandels auf Ihre Gesundheit informiert werden?*** | - Ja, sehr gerne - Eher ja - Egal - Eher nein - Nein, auf keinen Fall |
| --- | --- |
| **Möchten Sie von Ihrem Arzt/Ihrer Ärztin darüber informiert zu werden, …** |  |
| **D1b) … wie sich das Auftreten von Hitzewellen auf Ihre Gesundheit auswirken kann?** | - Ja, sehr gerne - Eher ja - Egal - Eher nein - Nein, auf keinen Fall |
| **D1c) …wie sich die Pollenflugsaison und das Auftreten von Allergien verändert und welche Folgen dies für Ihre Gesundheit haben kann?** | - Ja, sehr gerne - Eher ja - Egal - Eher nein - Nein, auf keinen Fall |
| **D1d) … wie sich das Auftreten von Infektionskrankheiten verändert und sich auf Ihre Gesundheit auswirken kann?** | - Ja, sehr gerne - Eher ja - Egal - Eher nein - Nein, auf keinen Fall |
| **Sollten Ihnen noch weitere Themen wichtig sein, über die sie in diesem Kontext gerne informiert werden würden, können Sie diese gerne hier angeben.** | ***(Freitextfeld)*** |

*Einige Menschen sind über den Klimawandel und andere Umweltveränderungen besorgt.*

| **D2a) Möchten Sie mit Ihrem Arzt/Ihrer Ärztin über Ihre Ängste oder Sorgen bezüglich der Auswirkungen des Klimawandels sprechen?** | - Ja, sehr gerne - Eher ja - Egal - Eher nein - Nein, auf keinen Fall - Nicht zutreffend |
| --- | --- |
| **D2b) Möchten Sie mit Ihrem Arzt/Ihrer Ärztin darüber sprechen, wie bzw. wo Sie sich für Klimaschutz engagieren können?** | - Ja, sehr gerne - Eher ja - Egal - Eher nein - Nein, auf keinen Fall |

*Weiter geht es mit Fragen zur Beratung bezogen auf mögliche Anpassungen an die gesundheitlichen Auswirkungen des Klimawandels.*

| **D3a) Möchten Sie von Ihrem Arzt/Ihrer Ärztin dazu beraten werden, wie Sie Ihre Gesundheit vor den Auswirkungen des Klimawandels schützen können?*** | - Ja, sehr gerne - Eher ja - Egal - Eher nein - Nein, auf keinen Fall |
| --- | --- |
| **Möchten Sie von Ihrem Arzt/Ihrer Ärztin dazu beraten zu werden, …** |  |
| **D3b) … wie ich Sie Ihre Gesundheit und die Ihrer Angehörigen an heißen Tagen und in Hitzewellen schützen können?** | - Ja, sehr gerne - Eher ja - Egal - Eher nein - Nein, auf keinen Fall |
| **D3c) … wie Sie sich vor klimawandelbedingten Veränderungen von Allergien und der Pollenflugsaison schützen können?** | - Ja, sehr gerne - Eher ja - Egal - Eher nein - Nein, auf keinen Fall |
| **D3d) … wie Sie sich vor klimawandelbedingten Veränderungen bei der Ausbreitung von Infektionskrankheiten schützen können?** | - Ja, sehr gerne - Eher ja - Egal - Eher nein - Nein, auf keinen Fall |
| **Sollten Ihnen noch weitere Themen wichtig sein, zu welchen Sie gerne in diesem Kontext beraten werden würden, können Sie diese gerne hier angeben.** | ***(Freitextfeld)*** |

*Der Klimawandel hat Einfluss auf die Menschen vor Ort, aber auch weltweit. Bitte teilen Sie uns Ihre Meinung zu folgenden Fragen mit.*

| **D4a) Möchten Sie mit Ihrem Arzt/Ihrer Ärztin Zusammenhänge zwischen Klimawandel und Gesundheit besprechen, die Ihre persönliche Gesundheit betreffen?** | - Ja, sehr gerne - Eher ja - Egal - Eher nein - Nein, auf keinen Fall |
| --- | --- |
| **D4b) Möchten Sie mit Ihrem Arzt/Ihrer Ärztin Zusammenhänge zwischen Klimawandel und Gesundheit besprechen, welche die Gesundheit aller Menschen betreffen?** | - Ja, sehr gerne - Eher ja - Egal - Eher nein - Nein, auf keinen Fall |

*Gesunde Lebensstile sind häufig auch gut für das Klima. Wie Menschen essen, hat Einfluss auf ihre Gesundheit und den Klimawandel. Auch die Art und Weise, wie Menschen sich tagtäglich fortbewegen und ob sie sich der Natur verbunden fühlen, kann Auswirkungen auf ihre Gesundheit und den Klimawandel haben.*

| **D5a) Möchten Sie mit Ihrem Arzt/Ihrer Ärztin darüber sprechen, wie Sie gesund und klimafreundlich leben können?*** | - Ja, sehr gerne - Eher ja - Egal - Eher nein - Nein, auf keinen Fall |
| --- | --- |
| **Möchten Sie mit Ihrem Arzt/Ihrer Ärztin darüber sprechen, …** |  |
| **D5b) … wie Sie sich gesünder und klimafreundlicher ernähren können?** | - Ja, sehr gerne - Eher ja - Egal - Eher nein - Nein, auf keinen Fall |
| **D5c) … wie Sie sich in Ihrem Alltag gesünder und klimafreundlicher fortbewegen können?** | - Ja, sehr gerne - Eher ja - Egal - Eher nein - Nein, auf keinen Fall |
| **D5d) … wie Sie durch Aufenthalt in der Natur Ihr seelisches Wohlbefinden verbessern können?** | - Ja, sehr gerne - Eher ja - Egal - Eher nein - Nein, auf keinen Fall |

*Nicht nur im Gespräch kann der Zusammenhang zwischen Klimawandel und Gesundheit ein Thema sein. Auch andere Informationswege sind möglich.*

| **E) Welche Informationsangebote seitens eines Arztes/einer Ärztin halten Sie für geeignet, um über die Zusammenhänge zwischen Klimawandel und Gesundheit informiert zu werden? Eine Mehrfachauswahl ist möglich.** | - Persönliches Gespräch - Flyer und Broschüren in der Praxis - Plakate in der Praxis - Internetauftritt der Praxis - Newsletter der Praxis - Social Media-Auftritt der Praxis - Ich halte keine dieser Informationsangebote für geeignet. |
| --- | --- |

*Sie haben nun mögliche Themengebiete kennengelernt, die im Rahmen einer klimasensiblen Gesundheitsberatung eine Rolle spielen können. Bitte beantworten Sie dazu zusammenfassend die folgende Frage.*

| **F) Möchten Sie, dass die Themen Klimawandel und Gesundheit im Gespräch mit ihrem Arzt/Ihrer Ärztin vorkommen?*** | - Ja, sehr gerne - Eher ja - Egal - Eher nein - Nein, auf keinen Fall |
| --- | --- |

*Es folgen nun noch zwei abschließende Fragen, welche die statistische Auswertung erleichtern sollen.*

| **G) Viele Leute verwenden die Begriffe „links“ und „rechts“, wenn es darum geht, unterschiedliche politische Einstellungen zu kennzeichnen. Wir haben hier einen Maßstab, der von links nach rechts verläuft.**  **Wenn Sie an Ihre eigenen politischen Ansichten denken, wo würden Sie diese Ansichten auf dieser Skala einstufen?*** | - Links - Rechts |
| --- | --- |
| **H) Wie oft waren Sie in den letzten zwölf Monaten ungefähr beim Arzt (Hausarzt oder Facharzt), um sich selbst behandeln oder beraten zu lassen?*** | - Gar nicht - 1 Mal - 2 Mal - 3 bis 5 Mal - 6 bis 10 Mal - 11 bis 20 Mal - Über 20 Mal - Möchte ich nicht angeben |

*Sie sind jetzt am Ende des Fragebogens angelegt. Wir bedanken uns herzlich für Ihre Teilnahme!*

*Falls Sie noch Anmerkungen zu diesem Fragebogen haben, können Sie diese hier mitteilen:*

| ***(Freitextfeld)*** |
| --- |

**Questionnaire 3.2.: English translation**

(*marks mandatory items)

*Introduction:*

*There are various links between climate change and health. We want to find out whether these should also play a role in the physician-patient conversation. We are interested in your opinion on this.*

*Now follow some questions about your attitude on climate change.*

| **Aa) How important is the issue of climate change to you personally?*** | - Extremely important - Very important - Somewhat important - Not too important - Not at all important |
| --- | --- |
| **Ab) How worried are you about climate change?*** | - Very worried - Somewhat worried - Not very worried - Not at all worried |
| **Ac) How much do you think climate change will harm you personally?*** | - A great deal - A moderate amount - Only a little - Not at all - Don't know |
| Ad) How much do you think climate change will harm future generations of people?* | - A great deal - A moderate amount - Only a little - Not at all - Don't know |

*The following questions deal with how important it is for you to behave climate-friendly.*

| **Ba), Do you make an effort to reduce your greenhouse gas emissions in your everyday life (i.e. your climate-damaging emissions)?** | - Yes, very - Rather yes - Partly - Rather no - Not at all |
| --- | --- |
| **How willing are you ...?** |  |
| **Bb) ... to use a bicycle/e-bike instead of a combustion vehicle in everyday life?** | - Very willing - Rather willing - Partly - Rather not willing - Not at all willing - Not applicable |
| **Bc) ... to use climate-friendly public transport (e.g. bus, train) instead of a car or plane in everyday life and for travel?** | - Very willing - Rather willing - Partly - Rather not willing - Not at all willing - Not applicable |
| **Bd) ... to eat a low meat diet? (i.e. little meat and cold meat). If you already eat fully vegetarian, select "Not applicable".** | - Very willing - Rather willing - Partly - Rather not willing - Not at all willing - Not applicable |
| **Be) ... to eat a completely vegetarian or vegan diet? (i.e. abstain from meat and cold meat or completely abstain from animal products)** | - Very willing - Rather willing - Partly - Rather not willing - Not at all willing - Not applicable |
| **Bf) ... to volunteer in an organisation or association for climate action?** | - Very willing - Rather willing - Partly - Rather not willing - Not at all willing - Not applicable |

*The following are questions about climate-sensitive health counselling with your physician. Climate-sensitive health counselling means that climate change and health topics are part of the medical consultation.*

| **Ca) In your opinion, has this kind of content on climate change and health ever been the subject of a consultation with your physician?*** | - Yes, often - Yes, at least once - No - Don’t know |
| --- | --- |
| **Cb) Please select whether the following topics have already been addressed by a physician of yours. You are also welcome to tick "None of these topics" or use the free text field to add something.*** | - Health risks from heat waves due to climate change - Connection between climate change and allergies - Connection between climate change and the physical consequences of extreme weather events - Impairment of mental health due to extreme weather events as a result of climate change - Connection between climate change and infectious diseases - Impairment of mental health due to concerns about climate change - Healthy and climate-friendly nutrition - Healthy and climate-friendly mobility - Spending time in nature to improve mental well-being - None of these topics - Other: (free text box) |

*Climate change affects our health in different ways in Germany. In the following questions you will learn about different impacts, which have all been scientifically researched. You can now indicate which topics would interest you in the context of climate-sensitive health counselling. Take special account of the fact that these topics would come up in a conversation with your physician.*

| **D1a) Would you like your physician to inform you about the health impacts of climate change on your health?*** | - Yes, very much - Rather yes - Doesn't matter - Rather no - No, not at all |
| --- | --- |
| **Would you like your physician to inform you about...** |  |
| **D1b) ... how the occurrence of heat waves can affect your health?** | - Yes, very much - Rather yes - Doesn't matter - Rather no - No, not at all |
| **D1c) ... how the pollen season and the occurrence of allergies are changing and what consequences this may have for your health?** | - Yes, very much - Rather yes - Doesn't matter - Rather no - No, not at all |
| **D1d) ... how the occurrence of infectious diseases is changing and may affect your health?** | - Yes, very much - Rather yes - Doesn't matter - Rather no - No, not at all |
| **If there are any other topics that are important to you that you would like to be informed about in this context, please feel free to indicate them here.** | ***(free text box)*** |

*Some people are concerned about climate change and other environmental changes.*

| **D2a) Would you like to talk to your physician about your fears or concerns regarding the impacts of climate change?** | - Yes, very much - Rather yes - Doesn't matter - Rather no - No, not at all - Not applicable |
| --- | --- |
| **D2b) Would you like to talk to your physician about how or where you can get involved in climate action?** | - Yes, very much - Rather yes - Doesn't matter - Rather no - No, not at all |

*We continue with questions on counselling on possible adaptation measures to the health impacts of climate change.*

| **D3a) Would you like your physician to advise you on how to protect your health from the effects of climate change?*** | - Yes, very much - Rather yes - Doesn't matter - Rather no - No, not at all |
| --- | --- |
| **Would you like your physician to advise you on...?** |  |
| **D3b) … how to protect your health and that of your loved ones on hot days and in heat waves?** | - Yes, very much - Rather yes - Doesn't matter - Rather no - No, not at all |
| **D3c) … how to protect yourself from climate change-induced changes in allergies and the pollen season?** | - Yes, very much - Rather yes - Doesn't matter - Rather no - No, not at all |
| **D3d) … how you can protect yourself from climate change-related changes in the spread of infectious diseases?** | - Yes, very much - Rather yes - Doesn't matter - Rather no - No, not at all |
| **If there are any other topics that are important to you and on which you would like to receive advice in this context, please feel free to indicate them here.** | ***(Free text box)*** |

*Climate Change impacts people locally and globally. Please indicate your opinion to the following questions.*

| **D4a) Would you like to discuss the links between climate change and the effects on your personal health with your physician?** | - Yes, very much - Rather yes - Doesn't matter - Rather no - No, not at all |
| --- | --- |
| **D4b) Would you like to discuss the links between climate change and the health of all people with your physician?** | - Yes, very much - Rather yes - Doesn't matter - Rather no - No, not at all |

*Healthy lifestyles are often also good for the climate. How people eat has an impact on their health and climate change. The way people get around every day and whether they feel connected to nature can also have an impact on their health and climate change.*

| **D5a) Would you like to talk to your physician about how you can lead a healthy and climate-friendly lifestyle?*** | - Yes, very much - Rather yes - Doesn't matter - Rather no - No, not at all |
| --- | --- |
| **Would you like to talk to your physician about ...** |  |
| **D5b) … how you can eat healthier and more climate-friendly?** | - Yes, very much - Rather yes - Doesn't matter - Rather no - No, not at all |
| **D5c) … how you can get around in your everyday life in a healthier and more climate-friendly way?** | - Yes, very much - Rather yes - Doesn't matter - Rather no - No, not at all |
| **D5d) … how you can improve your mental well-being by spending time in nature?** | - Yes, very much - Rather yes - Doesn't matter - Rather no - No, not at all |

*The connection between climate change and health cannot only be addressed in a conversation. Other information channels are also possible.*

| **E) Which information offered by your physician do you consider suitable for being informed about the connections between climate change and health? Multiple selection is possible.** | - personal conversation - flyers/booklets in the physician’s office - posters in the practice - internet presence of the practice - newsletter of the practise - social media presence of the practise - none of the above information chanelles are suitable. |
| --- | --- |

*Y**ou have now learned about possible topics that can be part of climate-sensitive health counselling. Please answer the following summary question.*

| **F)** **Would you like topics of climate change and health to be part of the consultation with your physician?*** | - Yes, very much - Rather yes - Doesn't matter - Rather no - No, not at all |
| --- | --- |

*The following two concluding questions are intended to facilitate the statistical evaluation.*

| **G) Many people use the terms "left" and "right" when referring to different political tendencies. Here is a scale that goes from left to right.**  **When you think of your own political views, where would you rank those views on this scale?*** | - Left - Right |
| --- | --- |
| **H) How often have you visited a physician (general practitioner or specialist) for treatment or advice in the last twelve months?*** | - None - Once - 2 visits - 3 to 5 times - 6 to 10 visits - 11 to 20 visits - more than 20 visits - Do not want to specify |

*You have now reached the end of the questionnaire. Thank you very much for your participation!*

*If you have any comments on this questionnaire, you can share them here:*

| ***(Free text box)*** |
| --- |
